# Biogenic Metal Oxides

**DOI:** 10.3390/biomimetics5020029

**Published:** 2020-06-23

**Authors:** Hipassia M. Moura, Miriam M. Unterlass

**Affiliations:** 1 Institute of Materials Chemistry, Vienna University of Technology, 1060 Vienna, Austria; hipassia.moura@tuwien.ac.at; 2 Institute of Applied Synthetic Chemistry, Vienna University of Technology, 1060 Vienna, Austria; 3 CeMM Research Center for Molecular Medicine of the Austrian Academy of Sciences, 1090 Vienna, Austria

**Keywords:** metal oxides, biominerals, bio-inorganic chemistry, silica, iron oxide, manganese oxide

## Abstract

Biogenic metal oxides (M_x_O_y_) feature structures as highly functional and unique as the organisms generating them. They have caught the attention of scientists for the development of novel materials by biomimicry. In order to understand how biogenic M_x_O_y_ could inspire novel technologies, we have reviewed examples of all biogenic M_x_O_y_, as well as the current state of understanding of the interactions between the inorganic M_x_O_y_ and the biological matter they originate from and are connected to. In this review, we first summarize the origins of the precursors that living nature converts into M_x_O_y_. From the point-of-view of our materials chemists, we present an overview of the biogenesis of silica, iron and manganese oxides, as the only reported biogenic M_x_O_y_ to date. These M_x_O_y_ are found across all five kingdoms (bacteria, protoctista, fungi, plants and animals). We discuss the key molecules involved in the biosynthesis of M_x_O_y_, the functionality of the M_x_O_y_ structures, and the techniques by which the biogenic M_x_O_y_ can be studied. We close by outlining the biomimetic approaches inspired by biogenic M_x_O_y_ materials and their challenges, and we point at promising directions for future organic-inorganic materials and their synthesis.

## 1. Introduction

Living organisms—from bacteria to plants and mammals—have developed functional materials to optimize their life. Functions that materials in living beings provide are, e.g., mechanical support and protection, light-harvesting (e.g., for photosynthesis), and navigation. While living beings are for the largest part composed of organic matter, quite a range of functional materials in living beings are of inorganic nature and recognized as of biogenic origin. The term ‘biogenic’, from Greek *bíos* (life) + *génos* (origin), means produced by living organisms. For instance, both certain bacteria, and bees use magnetic iron oxide particles as part of their navigation systems [1,2]. Another example are silica (SiO_2_) structures that are manufactured by diatoms, marine sponges, and plants, for protection of the organism [3]. The two latter examples of inorganic materials in living beings, Fe_3_O_4_ and SiO_2_, are both biogenic metal oxides. Aside from metal oxides, one finds other inorganic compounds in living beings. Yet, the overall number of biogenic inorganic compounds is very limited. To date, not more than a few dozens of inorganic materials have been identified in living beings [4]. The main inorganic materials found in organisms are metal carbonates, phosphates, halides, oxalates, sulfates, sulfides, and oxides [5]. Nearly all of these compounds belong to the realm of minerals, which are defined as being (*i*) crystalline, (*ii*) inorganic, and (*iii*) naturally occurring materials. When minerals are occurring as part of living beings, they are named ‘biominerals’. Minerals that are part of living beings are very much affected by their environment, e.g., the presence of biomolecules such as proteins and sugars affect crystallite sizes and shapes, which in turn has drastic effects on the final materials properties. The term ‘biominerals’ nicely differentiates them from minerals found in the Earth’s crust, which have often had thousands of years to crystallize under conditions such as high temperatures and pressures. The conditions and kinetics of growth of biominerals are very different, as living beings do not live for thousands of years, and even extreme thermophile organisms do not operate at temperatures beyond ~120 °C [6].

The pathways by which organisms precipitate biominerals and the functionality of the structures they fashion have been shaped by natural selection through geological time. Note that the conversion of available inorganic precursors to, for example, biogenic oxides structures could not have been performed before ~2.4 billion years ago, when the O_2_ content in the Earth significantly increased and more complex forms of life appeared [7]. Why are biominerals confined to a few dozens of compounds, that are mostly metal carbonates, phosphates, halides, oxalates, sulfates, sulfides, and oxides, and in fact mostly centered around a handful of metal cations, such as Ca^2+^, Fe^x+^, and Si^4+^? First, the elements found in the anions, such as CO_3_^2−^, SO_4_^2−^, or O^2−^, are not scarce. Element abundance is important for the different mineralizing species, as a species that would limit itself to scarce elements would not have the best chances of survival. Second, the latter compounds are typically highly water-insoluble. As living beings are water-based or even live in aqueous environments such as oceans, it is necessary that their protective shell or stability-giving skeleton does not dissolve in water. Additionally, the low water-solubility imparts the final biominerals to nucleate at high supersaturation, which eventually promotes the formation of many small crystallites. Small, mostly nano-sized, crystallites are in living beings often found in well-organized nanocomposites that, e.g., mechanically profit from the small crystallite sizes, and also allow for generating complex shapes even including curvature. Third, while the inorganic parts of many biominerals are typically highly water insoluble, they often exist as hydroxylated/hydrated intermediates [8]. The existence of such water-soluble intermediates is necessary so that the organisms can actually manipulate these precursors before they deposit the final biominerals at the desired place within the organism. Finally, both the involved ions can be nicely manipulated through living beings, which have a machinery (e.g., calcium-ion channels) that is well adapted to work with these species.

With this review, we give an overview of metal oxides (M_x_O_y_) found in living systems (note that while in many review articles metal oxides are often abbreviated as ‘MO’, we decided to herein use M_x_O_y_ to avoid confusion with ‘MO’ as common abbreviation of ‘molecular orbital’). Metal oxides are of particular interest in materials chemistry as, (*i*) they are useful for various applications, e.g., as magnetic materials, catalysts, or optical materials; and (*ii*) they can be synthesized by molecular bottom-up approaches (mostly notably through the sol-gel process). Important M_x_O_y_ are, for example, TiO_2_ (a powerful photocatalyst), Fe_3_O_4_ (a biocompatible magnetic material), and CeO_2_ (an important oxidative catalyst) [9]. The applications of M_x_O_y_ are significantly different to salt-type components in biominerals such as CaCO_3_, which are important components for, e.g., biomedical applications or construction. Structurally, the metal oxides found in living organisms are actually built up of covalent and/or iono-covalent bonds, which again is fundamentally different from salt-type biominerals. As (living) nature is one of the best sources of inspiration, we hope that reviewing metal oxides in living organisms might be of interest to materials scientists, chemists, physicists, and engineers, that are rather far away from biology. To enable an easy entry point for this broad range of disciplines, we have assumed a rather tutorial writing style. For allowing for accessibility of this review to chemists, materials and physicists scientists, we have taken case to explain the biological technical terms in footnotes. As material chemists, at the end of this review we are providing a perspective for materials chemistry.

While biominerals have been widely reviewed focusing on particular aspects, e.g., specific chemical or biological compositions [1,10,11,12,13]; evolutionary aspects [7]; organic-inorganic interfaces [14]; or with respect to some particular technological applications, such as the production of inorganic materials (e.g., ceramic oxides, semiconductors and metal nanoparticles) [15], we are not aware of any other review that exclusively focuses on metal oxides found in living systems and therefore hope to close this gap.

In the following, we will first introduce M_x_O_y_ and briefly describe how nature provides the starting compounds for building M_x_O_y_-based structures. Then, the diversity of M_x_O_y_ present in living nature (e.g., Si, Mn and Fe oxides) and their overall properties are discussed together with the organisms’ production techniques. Finally, we close with a perspective on the biomimetics of the biogenic M_x_O_y_ in materials chemistry, as well as the future directions we think materials chemistry inspired by the here reviewed systems might take.

### 1.1. Metal Oxides

Metal oxides are the most abundant materials in the Earth’s crust. They display high chemical stability. In an oxidative environment, such as the atmosphere of the Earth, oxides are the lowest free energy states for most metals, which explains their high chemical stability. Pure metals often develop an oxide layer. For instance, commercial aluminum foil is covered with a so-called ‘passivation layer’ of alumina (Al_2_O_3_). M_x_O_y_ are used in various applications for their optical, electrical, and magnetic properties, and in fact their properties span over a very wide range: their electronic properties span from semiconductors (e.g., ZnO or WO_3_) to insulators (SiO_2_); their optical properties span from optical transparency (SiO_2_) to high light absorption (Cu_2_O); and their magnetic properties span from diamagnetic (ZnO) to ferromagnetic (Fe_3_O_4_) [9,16].

**Figure 1:**
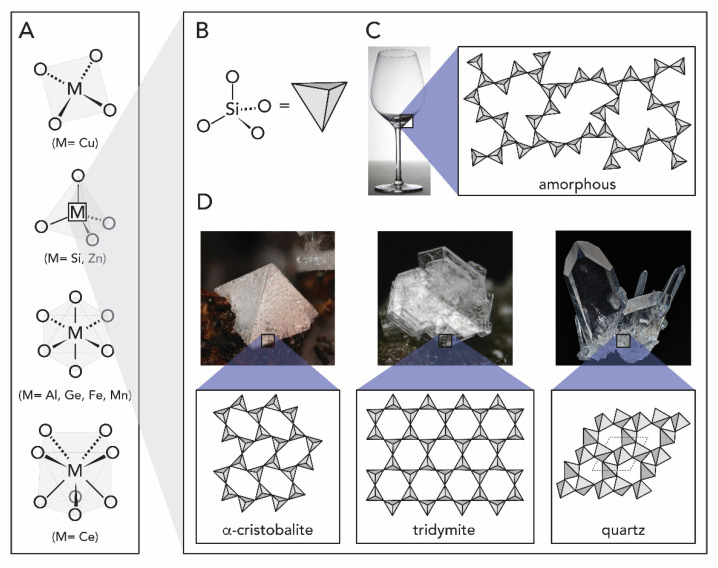
Different arrangements of metal oxides, featured silica and its forms. (**A**) Examples of coordination geometries that M_x_O_y_ can assume, from top to bottom: quadratic planar, tetrahedral, octahedral and square antiprismatic. (**B**) SiO_4_ tetrahedra in silica (SiO_2_). (**C**) Amorphous SiO_2_. (**D**) Examples of different polymorphs of crystalline SiO_2_: cristobalite, tridymite and quartz. Note that further crystalline polymorphs exist. Photographs of minerals are reproduced from www.mindat.org.

Chemically speaking, M_x_O_y_ comprise at least one type of metal and oxygen. Note that aside binary M_x_O_y_, numerous mixed metal oxides exist, e.g., FeTiO_3_. M_x_O_y_ can be subdivided according to the types of bonds that connect M and O, into (*i*) ionic, and (*ii*) covalent metal oxides. The type of bonding in a binary metal oxide is determined by the difference in electronegativity *∆EN* of the constituting metal and O, which is among the most electronegative elements (*EN*(O) ~3.4 (Pauling scale)). Very electropositive metals (e.g., Cesium with *EN*(Cs) ~0.8 (Pauling)) form salts (e.g., cesium forms the ionic oxide Cs_2_O at ∆*EN* ~2.6) [17]. Yet, metals of higher EN, such as the semimetal Si (*EN*(Si) ~1.9) form an ionocovalent (i.e., featuring strongly polarized covalent bonds) network [18]. In ionocovalent M_x_O_y_—and we will see later on that all biogenic M_x_O_y_ belong to that class—O is always bridging between two metal atoms. Depending on the stoichiometry, this can lead to several different coordination geometries of the metal (see Figure 1A), such as tetrahedral in SiO_2_, i.e., every Si is connected to for O, thus forming an SiO_4_ tetrahedral unit (Figure 1B), or, e.g., octahedral in Al_2_O_3_, thus forming AlO_6_ octahedra.

Silica, SiO_2_, occupies a special role amongst metal oxides in general as the most prototypical covalent M_x_O_y_, and also within the area of biogenic M_x_O_y_. Silica can be found in nature either in amorphous (Figure 1C) or crystalline forms (e.g., as quartz, cristobalite, tridymite, see Figure 1D). Crystalline silica, with quartz as the most well-known representative, is a common compound in soil, sand, and rocks, whereas amorphous silica is commonly deposited in living organisms, including plants, unicellular organisms such as diatoms, and multicellular organisms like sponges [3,19]. SiO_2_ notably also has one ‘inverse‘ pathway, from living beings to the ‘non-living’ nature: For instance, the mineral diatomite is in fact a sedimentary rock mainly consisting of the siliceous skeletal remains of diatoms [19].

Biogenic M_x_O_y_ span from amorphous representatives (SiO_2_) to crystalline ones (e.g., Fe_3_O_4_ and Mn_3_O_4_). Here, SiO_2_ is the exception: in general, amorphous representatives do not prevail in biology as the crystalline forms of a certain compounds are generally more soluble than their crystalline counterparts. In SiO_2_, this is overcome through a high energetic barrier to crystallization [20]. Biological organisms have evolved over billions of years to produce a wide variety of inorganic materials into diverse complex morphologies that are hierarchically structured on the nano-, micro- and macroscales. While in some cases fairly specific examples, the arrangements of M_x_O_y_ structures within living systems are in fact nice examples for the common phenomenon of hierarchically structured bio-materials in nature.

Amorphous SiO_2_ is most prominently found in diatoms, which are ornately shaped eukaryotic unicellular microalgae with cell walls made of a composite of organic material and silica. This living M_x_O_y_ system is extensively covered in the literature and the reader can find excellent references in a review by Hildebrand [10], for instance. Therefore, we have decided to exclude diatoms from this review article.

### 1.2. Origins of the Precursors of Biogenic Metal Oxides

**Figure 2:**
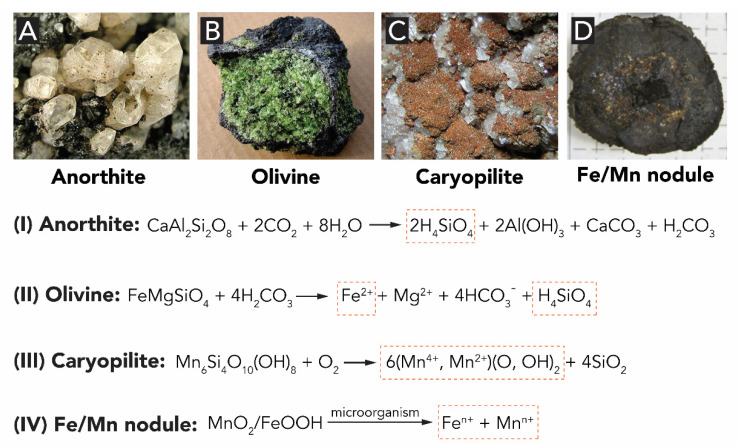
Representation of natural sources for the synthesis of metal oxides. (**A**–**C**) Examples of minerals that can suffer chemical weathering (reactions I-III) in subterraneous bedrock, releasing, e.g., iron, manganese and silicon biomineralization precursor species (as Si(OH)_4_; (Fe^2+/3+^) and (Mn^2+/4+^)(O,OH)_2_, highlighted by dotted boxes) to the aqueous environment. (**D**) The main sources of manganese minerals are found in the seafloor in the form of ferromanganese nodules, which release different ions through leaching. Photographs of anorthite by Rob Lavinsky, olivine by Aomai and caryopilite by Leon Hupperichs are published under Creative Commons Attribution (CC BY-SA 3.0) license. Photograph of ferromanganese nodule is reproduced from [23] with permission from the Elsevier B.V.

The Earth’s crust consists primarily (~90%) of silicates, i.e., minerals built up of anions of silicon and oxygen (i.e., [SiO$\begin{array}{l} \left(4-2x\right)\\ 4-x \end{array}$^−^]_n_ in, e.g., orthosilicate SiO_4_^4−^, metasilicate SiO_3_^2−^, and pyrosilicate Si_2_O_7_^6−^). Metal cations (e.g., Fe^2+^, Mn^2+^, Al^3+^ and Si^4+^) can be released from silicates by weathering (Figure 2A–C). Weathering describes the breakdown of igneous, metamorphic and sedimentary rocks, through the action of water, temperature and biological activity. The mechanical, microbiological as well as chemical weathering lead the transformation of mineral rocks to so-called mineral soil. The minerals present in the soil are chemically altered in this process (see reactions I–III in Figure 2), releasing water-soluble ions into their surroundings. Through water circulation in and above ground, these species are transported to different loci and later are taken up by living organisms (for instance, through the roots of plants) as we will see later.

The three biogenic metal oxides are SiO_2_, Fe_x_O_y_ (e.g., Fe_2_O_3_, Fe_3_O_4_ and α-/γ-FeOOH) and Mn_x_O_y_ (e.g., MnO_2_, Mn_2_O_3_ and their Ca, Na, and K mixed metal oxyhydrates todorokite (Ca,Na,K)_2_(Mn^3+/4+^)_6_O_12_·3-5H_2_O and birnessite (Na,Ca)Mn_7_O_14_⋅2-3H_2_O). Silica and iron oxide precursors mostly originate from the Earth’s crust, while for manganese oxides the situation is a bit different: Although Mn(II) is also released through the weathering of igneous and metamorphic rocks, which through oxidation results in more than 30 known Mn(III), Mn(IV), or mixed Mn(III,IV) oxide/hydroxide minerals, manganese (and ferromanganese) oxides are more commonly found as nodules and crusts on the ocean floor (Figure 2D). These nodules are typically brown-black spherical aggregates that consist of concentric layers of primarily MnO_2_ and iron oxide minerals (mostly FeOOH) [21,22]. For ferromanganese nodules, microbial oxidation is an important weathering process. In the following section, we summarize the origins of precursors for all three biogenic metal oxides: silica (SiO_2_), iron oxides (Fe_x_O_y_) and manganese oxides (Mn_x_O_y_).

#### 1.2.1. Silicon Oxide

**Figure 3:**
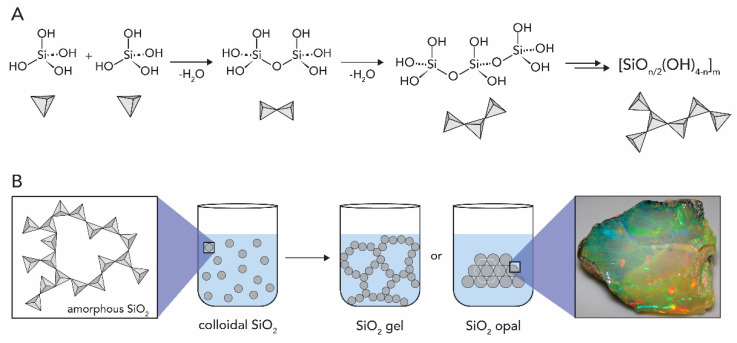
The silica formation. (**A**) Condensation of silicic acid Si(OH)_4_ to (hydrated) SiO_2_ polymers. (**B**) Silica gel and opal formation. Opal image by James St. John, published under Creative Commons Attribution (CC BY 2.0) license.

Silica is the most abundant M_x_O_y_ in living nature. During silicate weathering in the presence of CO_2_, ortho-silicic acid Si(OH)_4_ is released from the crystalline structure of silicate minerals (Figure 2A illustrates the weathering of anorthite to gibbsite as an example) [24]. In the environment, the Si(OH)_4_ dissolved in H_2_O is transported through the soil to rivers and the ocean. In water, at room temperature, Si(OH)_4_ and related species are stable for long periods of time at levels below ca. 100 ppm. However, once the concentration exceeds the solubility of the amorphous SiO_2_, around 100−200 ppm, it undergoes polycondensation reactions (see Figure 3A) [19]. Thus, silicic acid can precipitate as non-biogenic silica through self-condensation at >100–200 ppm. The precipitation of the silicic species generates silica (SiO_2_) which can be found either in amorphous (also known as opal, Figure 3B) or crystalline forms (e.g., quartz, cristobalite, tridymite, see Figure 1D). The variety and abundance of silicate minerals is a result of both the versatility and stability of silicon when bonded to oxygen, and the ease these Si_x_O_y_ structures incorporate other elements, forming a plethora of crystalline minerals [25]. Note that the geological weathering of the crystalline forms of silica requires millions of years.

The precipitation of biogenic SiO_2_ is fundamentally different from biominerals that are salts, such as CaCO_3_. The precipitation of salts is driven by solubility equilibria, while silica is an amorphous metal oxide formed by (inorganic) polymerization processes (Figure 3A). Amorphous SiO_2_, in contrast to its crystalline analogs, exhibits no long-range order. It is built up from SiO_4_ tetrahedra with Si-O-Si bonds, whose angles span over a broad range, and so do the Si-O bond distances. Amorphous SiO_2_ is a covalent inorganic polymer. Often the general formula [SiO_n/2_(OH)_4−n_]_m_ (where *n* = 0–4) is used to account for not fully condensed species. Amorphous SiO_2_ encompasses a broad variety of structural forms, from ordered opal aggregates to extended gel-like materials. Note that opal is built up of near-monodisperse spherical particles of hydrated silica (SiO_2_·nH_2_O) which are arranged in an ordered hexagonal particle lattice (see Figure 3). Opals are ‘mineraloids’ since they do not fulfill all three criteria of minerals (crystallinity, inorganic nature, natural origin) that we have seen previously with respect to crystallinity. When a Si(OH)_4_ solution is supersaturated, small particles are formed through condensation of monomers and small oligomers. Consequently, (hydrated) silica is precipitating until the supersaturation is relieved (Figure 3A). Colloidal particles of silica are formed through the growth of the initial nuclei via further polycondensation. These, in turn, may be aggregated to form a silica gel (little interparticle order) or an opal (SiO_2_ particle crystal), both of which bear surface Si-OH groups (see Figure 3B) [19].

In biological organisms, silica exhibits no long-range order, irrespective of if it is found in simple organisms, animals or plants. These materials are all formed under ambient temperature and pressure and at circumneutral pH. Yet, many of the species are able to produce robust hierarchical structures through a process called biosilicification.

A comprehensive definition for biosilicification, i.e., the process of generating biogenic silica, is as: “the movement of silicic acid from environments in which its concentration does not exceed its solubility (<2 mmol/L) to intracellular or systemic compartments in which it is accumulated for subsequent deposition as amorphous hydrated silica” [26]. Biosilicification is largely studied for diatoms and sponges, which usually involves organic biomolecules such as proteins, peptides and small amines in stages crucial to biosilica formation, such as Si uptake, transport and deposition. Combining approaches from molecular biology, genetics and cell biology, researchers have unveiled mechanistic aspects of biosilica formation in these systems. Examples include the identification of silicon transporters which are postulated to actively uptake silicic acid from the environment into the silicifying organisms [27], propylamines found in diatoms and sponges that have been shown to rapidly precipitate silica in in vitro in model reactions [28], and silicatein proteins from a range of silica sponges [29,30,31]. We are detailing the SiO_2_ formation on living organisms in Section 2.1, for the different SiO_2_-mineralizing species.

#### 1.2.2. Iron Oxides

Iron is released to the environment by the weathering of Fe^2+^-containing silicates (e.g., biotite, pyroxene, amphibole, olivine). Figure 2A illustrates the chemical weathering of the iron silicate olivine. Weathering of iron minerals is usually aided by microorganisms. Dissolved Fe^2+^ (so-called ‘ferrous’ iron) oxidizes spontaneously to Fe^3+^ (‘ferric’ iron), which in turn instantaneously reacts with water to form amorphous ferrihydrite (which is an iron oxyhydroxide, see Reaction 1) [32]. As we will see later, living organisms can transform these Fe^3+^ oxyhydroxides into crystalline structures. (see Reaction 1. Reaction equation of the oxidation of ferrous ions to ferric oxide):

4Fe^2+^ + O_2_ + 6H_2_O → 4FeOOH + 8H^+^

**Figure 4:**
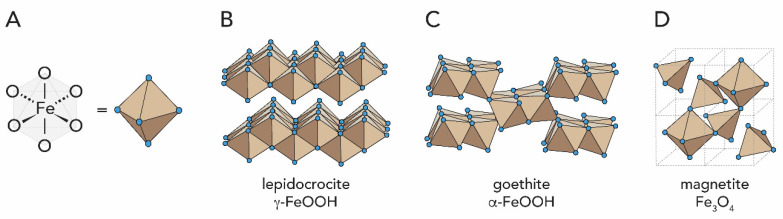
Examples of crystalline forms of iron oxides in living nature. (**A**) Octahedral geometry of iron. (**B**) Lepidocrocite, (**C**) goethite and (**D**) spinel structure of magnetite. For clarity, only oxygen is shown (blue sphere), while iron (not shown) is at the center of the polyhedral.

Among the crystalline forms of iron oxides, lepidocrocite (γ-FeOOH), goethite (α-FeOOH) and magnetite (Fe_3_O_4_) have been found in living species together with some amorphous oxy-hydroxides, Figure 4 (note that according to the Strunz classification, hydroxides (and oxyhydroxides) are considered oxide minerals [32]. Moreover, in accordance with Strunz Mineralogical Tables, we herein adopted the form FeOOH to represent oxyhydroxides instead of the sometimes also used FeO(OH)). The most studied iron biomineral is magnetite (Figure 4D), which has a cubic, inverse spinel structure and is ferrimagnetic, i.e., it presents permanent magnetism, at ambient temperature [33]. Their incorporation into living organisms will be discussed in Section 2.3.

#### 1.2.3. Manganese Oxides

**Figure 5:**
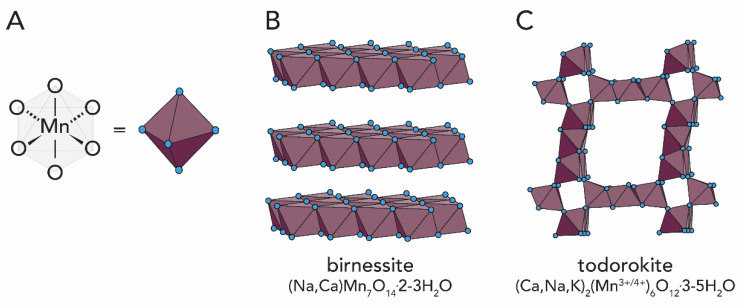
Examples of crystalline forms of manganese oxides in living nature. (**A**) Octahedral geometry of manganese. Representation of layered birnessite (**B**) and tunnel-type todorokite (**C**) structures. For clarity, only oxygen is shown (blue sphere), while Mn (not shown) is situated at the center of the octahedra.

Mn is released by weathering of Mn^2+^-containing silicates (e.g., biotite, pyroxene, amphibole) and leaching of ferromanganese nodules. As for the Fe oxides, the weathering of Mn minerals is heavily supported by microbial activity. Bacteria catalyze the oxidation of Mn^2+^ by indirect and direct processes. Indirect oxidation of Mn^2+^ occurs when organisms modify the pH and/or redox conditions of the local aqueous environment, or by the release of metabolic products that chemically oxidize Mn^2+^. In the direct process, bacteria oxidize Mn^2+^ through the production of polysaccharides or proteins [21]. The elucidation of the biochemical process involved in Mn^2+^ oxidation has only been recently achieved and will be detailed later on. Biogenic manganese oxides are mainly found in bacteria and fungi, and occur in many cases as crystalline layered birnessite ((Na,Ca)Mn_7_O_14_·2-3H_2_O) or tunnel-type todorokite ((Ca,Na,K)_2_(Mn^3+/4+^)_6_O_12_·3-5H_2_O); see Figure 5, further discussed in Section 2.4.

## 2. The Biogenesis of Metal Oxides

Silica is the second most abundant M_x_O_y_ biomineral after carbonates. Related ferric oxide minerals rank in the fourth most extensively formed biogenic M_x_O_y_. Manganese oxides are much less abundant than Si and Fe oxides, and are mainly found in bacteria and fungi.

**Figure 6:**
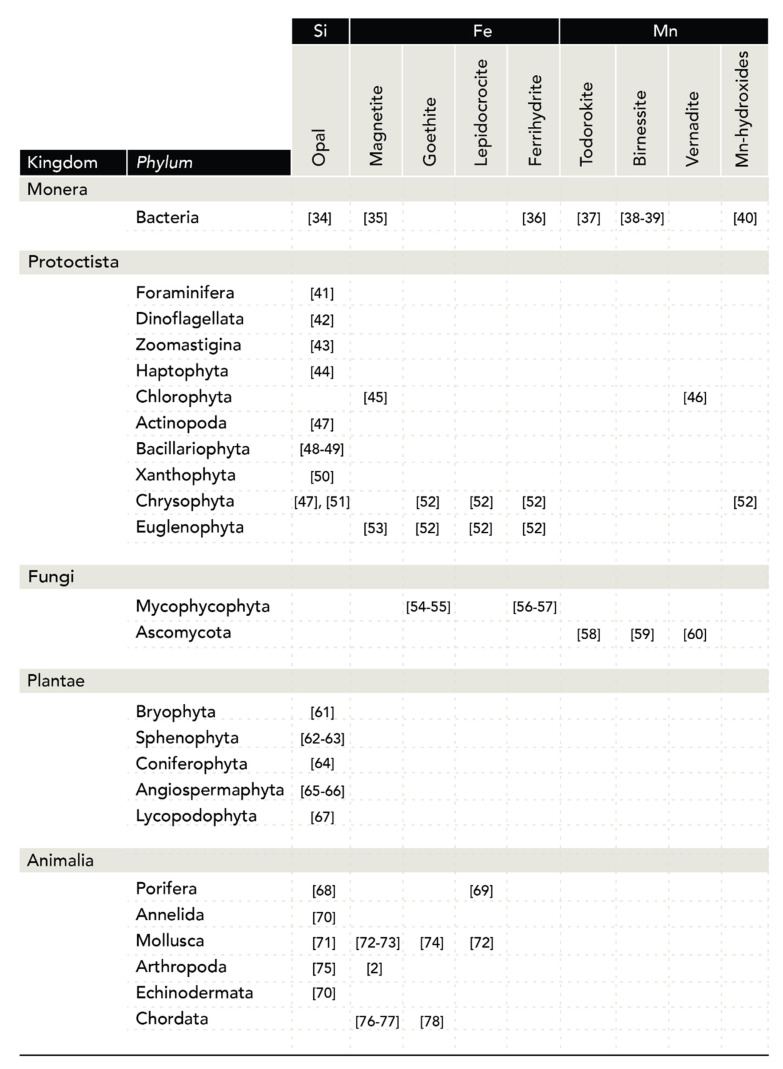
Location of biogenic metal oxides in the five different kingdoms. References are given in angular brackets. Compiled using data from references [2,34,35,36,37,38,39,40,41,42,43,44,45,46,47,48,49,50,51,52,53,54,55,56,57,58,59,60,61,62,63,64,65,66,67,68,69,70,71,72,73,74,75,76,77,78].

Figure 6 lists examples of biogenic M_x_O_y_. The table is organized according to their distribution in the phyla of the five kingdoms [5]. For specific taxa in certain phyla, please consult [8]. From Figure 6, it becomes clear that silica and iron oxides are the most representative M_x_O_y_ in living nature and are widely distributed among the kingdoms. In terms of structures formed, opal (amorphous SiO_2_) is clearly the most abundant, followed by iron oxides, particularly magnetite.

### 2.1. Silicon Dioxide

Biogenic silicas are the product of biological processes that lead to the formation of composite materials of diverse composition, hierarchical structures, and functions. SiO_2_ is found in various organisms ranging from bacteria to plants. In the following, we will focus on examples of silica present in bacteria and fungi, plantae and animalia kingdoms.

#### 2.1.1. Bacteria (Kingdom *Monera*)

Microbes, irrespective of where they live, commonly act as templates for the nucleation and precipitation of amorphous silica (opal-A) and other minerals through the reactive sites that exist on their surfaces [79]. Microbial mineralization is commonly associated with hydrothermal vents on land, lake floors, and the seafloor. Here, the waters originating from deep hot reservoirs containing besides other metallic species, dissolved silicic acids. When these fluids are discharged at the surface, rapid cooling to ambient temperatures, evaporation, and changes in the pH of the solutions cause the sudden surpassing of the solubility of amorphous SiO_2_. This prompts monomeric ortho-silicic acid, Si(OH)_4_, to polymerize, initially to oligomers and then to polymeric species, which further precipitate as amorphous hydrated silica. These freshly precipitated (hydrated) SiO_2_ species are commonly referred to as ‘sinters‘ [80].

**Figure 7:**
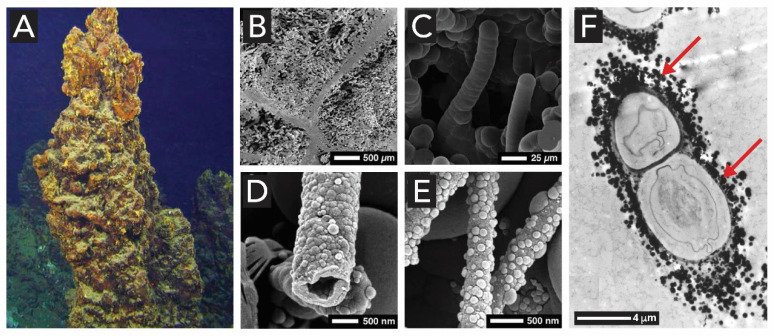
Silicified microbes. (**A**) Underwater photographs of silica-rich chimneys (~2 m) on the Giggenbach volcano. (**B**–**E**) SEM photomicrographs of silicified microbes: (**B**) Overview of silicified microbial mats with opal-A sheets. (**C**) Silicified filamentous microbe. (**D**) Silicified rod-shaped microbe showing a thin wall around an open lumen and small opal-A spheres on the outer surface. (**E**) Silicified rod-shaped microbes coated with opal-A and scattered opal-A spheres. (**F**) TEM cut of silicified bacteria from a geyser outflow channel, showing a filamentous cyanobacterium with silica spheroids on outer sheath (arrows). Images A–E are adapted from [79] with permission from John Wiley and Sons. Image F is reproduced from [80] with permission from Springer Nature BV.

Most siliceous sinters contain silicified microorganisms. For example, Jones et al. have reported silicified microbe mats (Figure 7B) found in chimneys of submarine volcanoes (Figure 7A), and analyzed these with respect to their morphology and biogenicity [79]. Although DNA analyses of the samples were inconclusive, the authors showed that the mineralized microbes in the samples are formed of at least three different types of opal-A morphologies (Figure 7C–E), i.e., filamentous microbes, rod-shaped microbes and spherical microbes. Complementarily, the work of Konhauser et al. on silicified bacteria from a geyser outflow channel shows microbial cells encrusted in spheroidal grains of silica extracellularly on the sheaths or walls of living cells (Figure 7F) [80].

**Figure 8:**
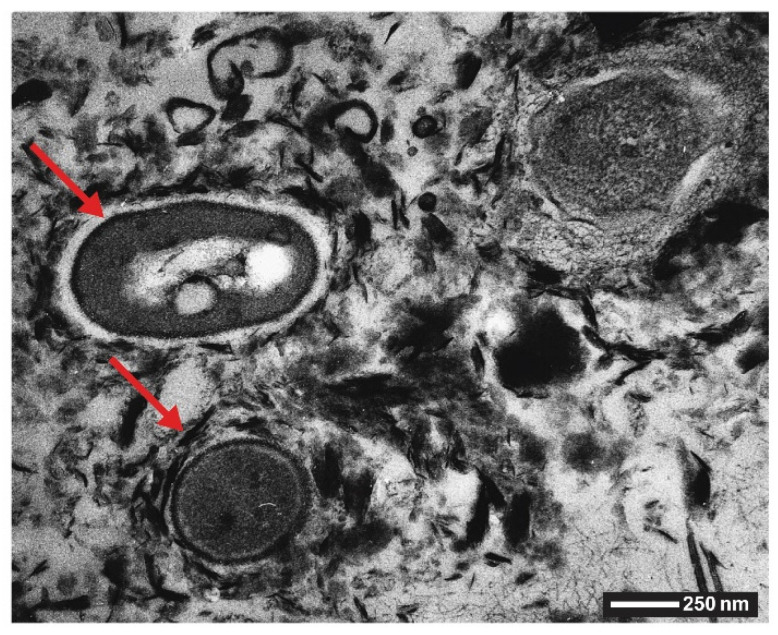
Electron microscopy image of mineral precipitates closely associated with bacterial cells from a microbial mat community in a saline alkaline lake. The thin, dark deposits on and around the cells (arrows) contain a Fe,Mg-silicate, resembling the mineral sepiolite. Scale bar: 250 nm. Reproduced from [84] with permission from Elsevier.

Moreover, a number of silicates associated with Fe and Al oxides of clay-type have been observed on the surface of bacterial envelopes in different environments. The metal content of the clays is usually reflecting the metals present in the surrounding water. In river sediments [81], hot springs [82], and mine tailings [83], silicate minerals of usually a clay-like composition and structure are found in association with bacteria [84]. The silicate deposits in these microbes are found as thin (~100 nm) crusts on and around the bacteria cells (Figure 8). It is likely that the bacteria play an important role in their formation, rather than simply binding pre-formed minerals detritus, since (*i*) the composition of silicates found on the cells differs markedly from those silicates not associated with bacterial cells, and (*ii*) the silicate precipitates found on the cell are more crystalline and larger in structure. Interestingly, even in environments with low Si(OH)_4_ concentrations, such as freshwater lakes and rivers, clay-like precipitates are found on bacterial cells.

A possible process for the formation of silica in this context involves the direct interaction of soluble anionic silicates with the positively charged groups on the peptidoglycan (a polymer of sugars and amino acids that shapes the cell wall) of the bacterial envelopes, such as the amine side groups on the peptide chain. Hydrogen bonding between silanol groups and the polysaccharide hydroxy groups of the cell wall may also be involved. If the superficial bacterial layers are predominantly negatively charged, external cations (Al, Fe) can interact with the cell wall and also provide nucleation sites for the mineralization [85,86]. A combination of these processes seems to be involved in the formation of silica at the surface of the Gram-positive bacterium *Bacillus subtilis* [85] and Gram-negative *Thiobacillusin* [83] mine tailings. This coating is often associated with extreme environments, which suggests that it may also serve as a protecting shell for the bacteria.

#### 2.1.2. Sponges (Kingdom *Animalia*, Phylum *Porifera*)

Sponges, members of the phylum *porifera*, are multicellular organisms that have porous and opened-channel silica bodies of a plethora of morphologies, that allows circulation of water through their bodies. Like diatoms, sponges produce a variety of skeletons and they are classified into three classes: *Hexactinellida*, *Desmospongiae* and *Calcarea*. Although all three utilize biomineralization in forming their skeletons, only *Hexactinellida* and *Desmospongiae* do so using silicon to build robust structures of silica while *Calcerea* utilize calcium carbonate [8]. The chemical composition of demosponge and hexactinellid spicules varies slightly depending on the species and the water composition in the habitat, but it is mostly silica (SiO_2_), and water, with some trace elements [87]. Sponges take up silicon in the form of soluble silicic acid to form spicules. Si uptake by sponges has been measured in laboratory experiments and may vary according to the Si concentration in the water, the temperature, and other environmental factors that affect sponge physiology and metabolism [29].

For providing a perspective for the M_x_O_y_ biominerals reviewed in this work, we are in the following summarizing the biomolecules involved in sponge silica formation. As we think that biosilicification process can serve as biomimetic inspiration for the synthesis of SiO_2_ (see perspective Section 3), we have decided to in the following summarize the involved process from a chemical/synthetic point of view. Note that the biosilicification processes of diatoms and sponges have been reviewed in great detail in [3,10,15,29,88,89].

**Figure 9:**
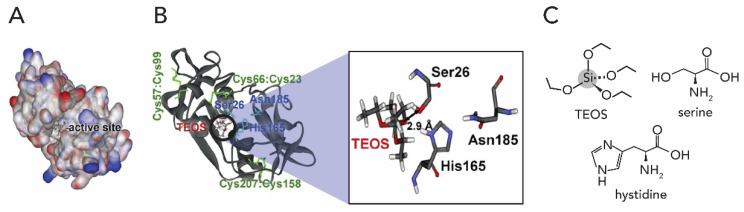
Model of silicatein-a from *S. domuncula*. (**A**) 3D model of the electrostatic charge distribution of silicatein-a (red: positive charges; blue: negative charges; white: hydrophobic areas). (**B**) The authors have co-crystallized tetraethylorthosilicate (TEOS) with silicatein, which as the crystal structure reveals is indeed found in the active site. Detail shows the steric proximity of the amino acids of the catalytic center to the TEOS molecule. (**C**) TEOS, histidine and serine molecules. Adapted from [30] with permission from Springer Nature.

A major breakthrough in the understanding of biosilicification of the silica-spicule, a sharp-pointed structure occurring in the skeleton of a sponge, was prompted through the discovery of a key enzyme. Morse et al. discovered that the organic filament in the central canal of the spicules is composed of a cathepsin L-related enzyme, which are termed silicatein (Figure 9) [90,91].

**Figure 10:**
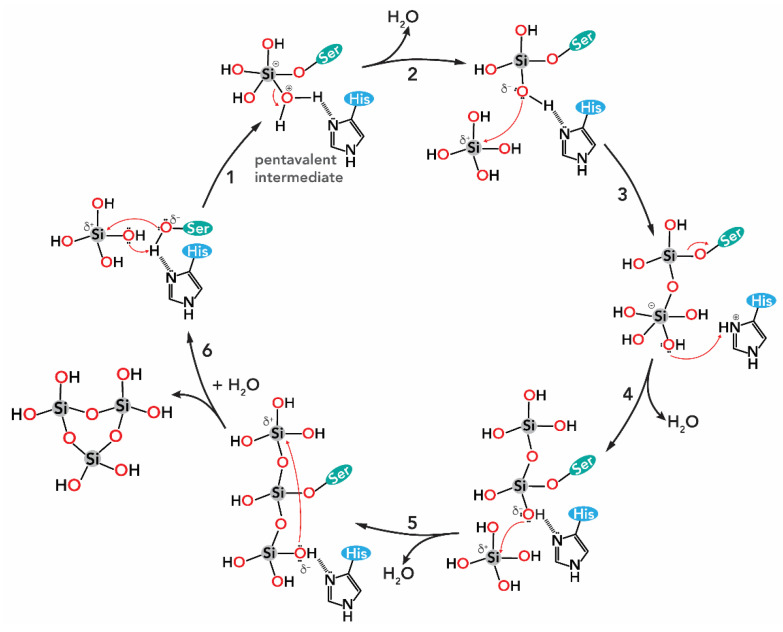
The proposed mechanism of silicatein-catalyzed polymerization of orthosilicic acid. Note that the example shown as product is a trisiloxane (SiO)_3_(OH)_6_. Adapted from [30] with permission from Springer Nature.

The mechanism of the silicatein catalyzed polymerization of Si(OH)_4_ to SiO_2_ relies on the assumption that the presence of both the serine and histidine residue in the catalytic centre of silicatein are essential for the interaction with the substrate orthosilicic acid and the subsequent polymerization. One of the proposed catalytic mechanisms is described in Figure 10 [30].

The following process proceeds in the catalytic pocket of silicatein (Figure 10): (*i*) in the first step, a nucleophilic attack (S_N_2-type) of the (electronegative) O of the serine residue’s OH group to the (electropositive) Si atom of a Si(OH)_4_ molecule (step 1). This mechanism is facilitated by the H-bond between the Ser and the His residues, which increases the nucleophilicity of the serine‘s OH group. Then, the H-transfer from the Ser–His H-bond to one of the OH ligands of Si(OH)_4_ occurs, forming a pentavalent intermediate and the serine residue is positioned in the catalytic centre of silicatein. (*ii*) After release of water (step 2), this serine-bound silicic acid molecule undergoes a nucleophilic attack at the silicon atom of a second Si(OH)_4_ molecule and generates a disilicic acid molecule (step 3) that is bound to silicatein. (*iii*) Then, the rotation of the Si-O-Ser bond is required to allow an interaction of a second OH ligand of the enzyme-bound silicic acid unit with the nitrogen imidazole of the catalytic centre His residue (step 4), facilitating further growth of the disilicic acid by nucleophilic attack to a third Si(OH)_4_ molecule (step 5). (*iv*) Several repetitions of the reaction cycle (*S_N_2*-type attack, H-transfer, loss of water, rotation) result in higher membered silicic acid oligomers. (*v*) The covalent linkage between the enzyme and the silicic acid oligomer is hydrolyzed by water (step 6), thus freeing the enzyme pocket.

**Figure 11:**
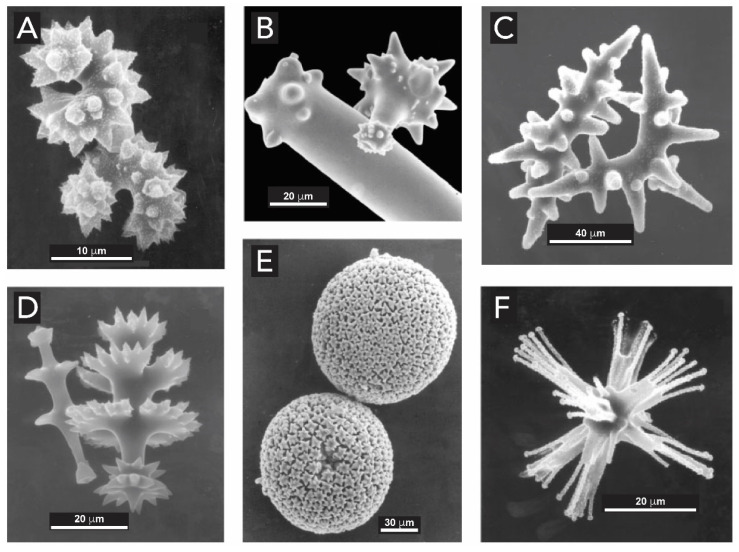
Scanning Electron Microscopy (SEM) images of spicules from different orders of *Demospongiae* and *Hexactinellida* sponges. (**A**) Spirasters of *Spirastrella*. (**B**) Asterose microacanthostyle of *Discorhabdella*. (**C**) Microscleres of *Paradesmanthus*. (**D**) Discorhabds of *Latrunculia*. (**E**) Sterrasters of *Geodia*. (**F**) Floricome (microhexaster) of *Sympagella*. (Spirasters: a short curved axial rod-like spicule with thick spines. Asterose: small star-shaped spicules. Microsclere: a flesh-spicule of a sponge, usually found as the support of a single cell. Discorhabds: a monaxial straight rhabd (rod-shape) with one sharp and one blunt end and girdled with concentric rings. Sterrasters: ball-shaped spicules. Floricome: branched hexaster spicules.) Reproduced from [87] with permission from John Wiley and Sons.

Spicules are of particular interest. These structures consist of glassy rods with diameters ranging from a few microns to several mm, and enormous lengths reaching up to 3 m [92]. They exhibit high fracture toughness and low elastic moduli, which leads to outstanding flexibilities for which there is no equivalent amongst synthetic silica-based materials. Spicules can present diverse morphologies as illustrated in Figure 11. They are used for structuring the skeleton, defense, food entrapment, and anchoring of the sponge on the seafloor. As it has been shown for diatoms, spicule synthesis and morphologies are both genetically encoded and influenced by environmental factors such as wave forces and local silicon concentrations [87].

**Figure 12:**
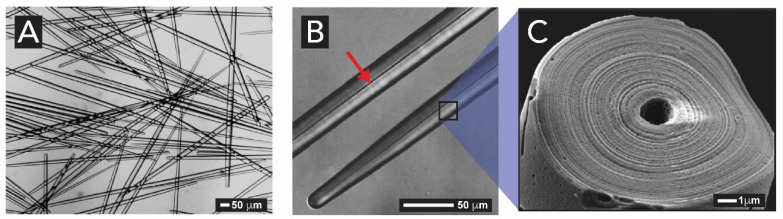
Isolated spicules from *Tethya aurantia.* (**A**) Optical micrographs of isolated spicules measuring 30 μm in diameter and 2 mm in length and (**B**) its higher magnification image showing axial filaments running through the center of the *T. aurantia* spicules (arrow). (**C**) Scanning electron micrographs of a cross-section, showing the concentric circular siliceous deposits after removal of the preexisting axial filaments. Scale bars: (A,B) 50 μm, (C) 1 μm. Adapted from [93] with permission from John Wiley and Sons.

Spicules are made of concentric layers of hydrated amorphous silica surrounding an axial proteic filament (Figure 12). This filament does not only template silica deposition but also catalyzes the polymerization of silicic acid species [87,93]. The morphological, physical, and chemical characteristics of spicules were extensively analyzed by Müller and coworkers, using optical and electron microscopy and high resolution nuclear magnetic resonance [29,30,89,94,95]. It could be demonstrated that spicule formation starts within specialized cells, the sclerocytes, around the axial filament. After the silica layer around the axial filament reaches a diameter of approximately 3–5 μm, this layer becomes surrounded by organic silicatein, which synthesizes the second siliceous layer, and this process is repeated many times until many concentric layers are formed. If the formation of siliceous spicules is inhibited, the sponge body collapses. Aside silicateins, other proteins such as galectin and silicase are believed to be necessary for the synthesis of spicules [96]. Galectin binds silicatein and forms an organized net-like matrix on the surface of the spicules, while silicase is able to depolymerize amorphous silica and thereby allows for corrections in the skeleton formation [97].

**Figure 13:**
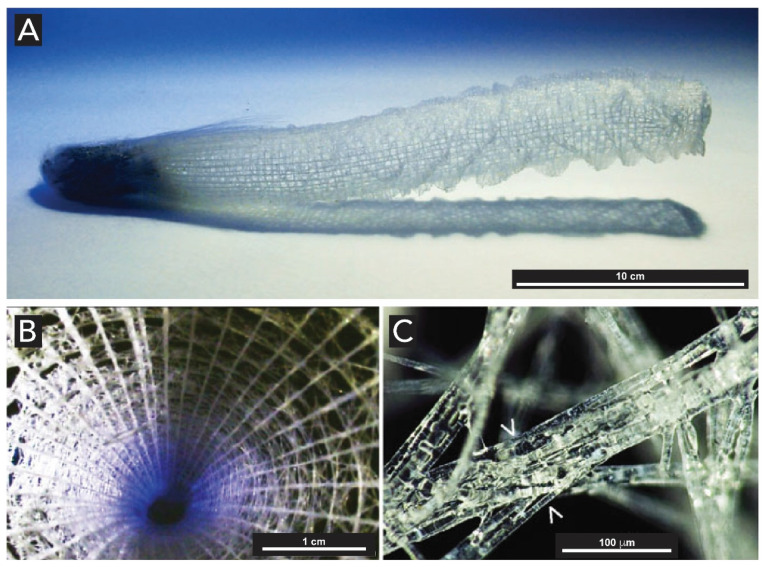
The skeleton of the *hexactinellid E. aspergillum* (**A**) is built of tightly interacting spicules (**B**). In (**C**) it is visible that those spicules even fuse together (indicated by >). Scale bar: (A) 10 cm, (B) 1 cm) and (C) 100 μm. Adapted from [100] under the Creative Commons Attribution 4.0 International License.

The spicule skeleton of sponges is of a highly complex architecture with the spicules being glued together by a collagenous ‘cement’ [98]. In rare cases, chitin is used instead of collagen as a binder [99]. The most beautiful examples of such complex architectures are glass-sponges (*hexactinellida*), e.g., in *Euplectella aspergillum* (Figure 13A). From the micrograph displayed in Figure 13B, taken inside the sponge skeleton, it can be seen that the spicule-network renders a well-defined shape. Their spicules grow to larger sizes and fuse to form a continuous super-scaffold (Figure 13C). These complex spicule frameworks are in fact used as a light transmission system, as described in the following section [100].

#### 2.1.3. Light-Harvesting in Sponges

The function of sponge spicules as optical waveguides was first described by Cattaneo-Vietti et al. in 1996 [101]. They showed that siliceous spicules from *Rossella racovitzae*, a *hexactinellid* species, act as light-collecting optical fibers. A decade later, Müller and coworkers showed that only light between 615 and 1310 nm (visible and near-infrared regions, respectively) can pass through the spicules of *hexactinellid Hyalonema sieboldii* [102], and spicules of *Monorhapsis sp* [103]. Although there is a substantial body of evidence that silica spicules present interesting optical properties, only few species exhibiting light harvesting and transmitting functions have been reported. It was found that sponge spicules can show long-lived fluorescence with a maximum at 770 nm and strong optical nonlinear behavior when pumped with femtosecond laser pulses [104]. Recently, a study concluded that the spicules of *Sericolophus hawaiicus* sponges might act as a natural supercontinuum generator (supercontinuum generation is a combination of nonlinear optical effects that spectrally broaden an initially nearly monochromatic laser beam, and it is used to provide intense sources of white light [105]), involving wavelengths between 650 and 900 nm [106]. This optical property was assumed to be possible through the spicules’ composite nature, i.e., have a combination of silica and an organic matrix. In fact, the speculations about a possible light reception system were confirmed by Müller and coworkers [100]. For the first time, Müller and coworkers reported that the demosponge *Suberites domuncula* exhibits a synergistic interaction of their siliceous spicule system with a biosensor system consisting of a light releasing luciferase. The light-generating luciferase protein has been identified, and it was shown that the luciferase together with the inorganic spicule acts as waveguides for the transmission of light and electrical and chemical signals, similar to a ‘neuron-like’ network system [103,107].

**Figure 14:**
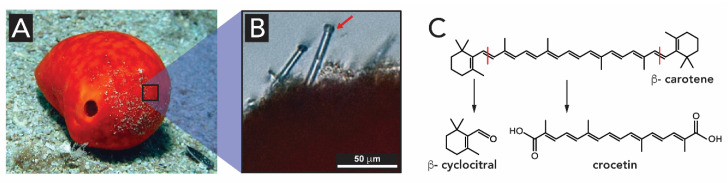
Light harvesting system in *S. domuncula.* (**A**) A specimen of the demosponge *S. domuncula* from the Adriatic Sea. (**B**) Detail of the surface of the sponges shows protruding spicules, tylostyles (uniradiate pointed sponge spicule with a knob at the blunt end) (arrow), which expose their spherical/elliptical knobs to the external environment, while the tips remain inside. Scale bar: 50 μm. (**C**) The carotenoids found in *S. domuncula*; they are the degradation products from b-carotene (identified as crocetin and b-cyclocitral). Image (**A**) by Anne Frijsinger and Mat Vestjens, 2004. Images (**B**,**C**) Adapted from [100] under the Creative Commons Attribution 4.0 International License.

Further investigations revealed that not only light generated within the interior of the sponges by luciferases might be present [108]. Other substances have been shown to be involved in light harvesting. The *S. domuncula* sponge (Figure 14A) displays spicules of 150–320 μm on their surface, with a globular swelling at one end and a pointy tip at the other end (Figure 14B). The authors assume that the globular siliceous ends act as convex lenses that focus the light. The authors identified the red colored compounds in the sponge tissues as carotenoids with spectral maxima at 400 and 430 nm. The reddish-yellow color of the sponge comes from the degradation products of β-carotene to b-cyclocitral and crocetin (Figure 14C). Sponges of type *S. domuncula* are assumed to collect the light within the spherical knobs of their spicules, and guide it through the spicules into the interior of the organism. The carotenoid pigments seem to play a dual role: (*i*) they are involved in certain metabolic activities, and (*ii*) act as a sensitizers and dopants for the silica-spicule light waveguide [100].

#### 2.1.4. Plants (Kingdom *Plantae*)

**Figure 15:**
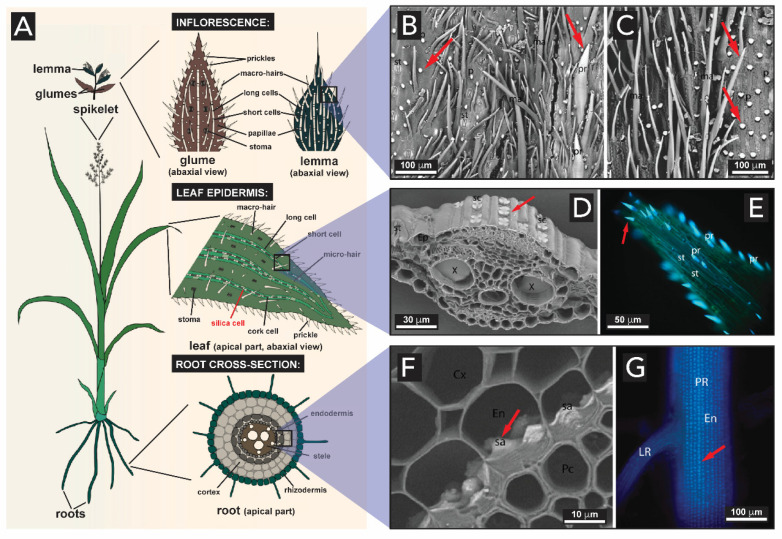
Silica deposition in grasses. (**A**) General scheme of a grass and typical silicification patterns in the inflorescence (top), leaf epidermis (middle), and root cross-section (bottom). White represents silicified cells. (**B**) Scanning electron micrograph (SEM) of the abaxial epidermis of glume in *Triticum aestivum L*. (**C**) SEM of the abaxial epidermis of lemma in *T. estivum*. (**D**) SEM of *Sorghum bicolor (L.) Moench* leaf cross-section showing silica cells in the epidermis. (**E**) Fluorescence micrograph of prickles at the leaf tip in *S. bicolor* visualized by alkali-induced fluorescence. (**F**) SEM of *S. bicolor* root cross-section showing silica aggregates anchored in the inner tangential cell walls of endodermis. (**G**) Alkali-induced fluorescence micrograph of *S. bicolor* primary root showing distribution of silica aggregates in the endodermis of the root. Red arrows highlight examples of silica bodies in the organs. SEMs were collected at the back scattered electron mode, rendering silicon atoms brighter than carbon atoms. (Cx: cortex; En: endodermis; Ep: epidermis; LR: lateral root; ma: macro-hair; p: papilla; Pc: pericycle; PR: primary root; pr: prickle cell; sa: silica aggregate; sc: silica cell; st: stoma). Adapted from [112], original by Kumar, Soukup and Elbaum, 2017, under the terms of the Creative Commons Attribution License (CC BY).

Terrestrial plants such as grasses, bamboo, and certain tropical trees, absorb large quantities of silicon from the soil and deposit it as hydrated amorphous silica in different tissues, from the roots to the stems and leaves (Figure 15A) [109,110,111]. In grasses, silica is deposited in all the organs, and the highest level of silicification is usually found in the inflorescence epidermis (Figure 15B–C), leaves (Figure 15D–E) and in the root (Figure 15F–G) [112]. Note the following definition: Inflorescence: the shoot of seed plants where flowers are formed, i.e., the reproductive portion of a plant. Glume: each of two bracts (a leaf-like structure) surrounding the grass flower (known as spikelet). Lemma: bracts at the base of each individual small flower (floret) clustered within an inflorescence. See detail in Figure 15A.

SiO_2_ structures are found in the inflorescence bracts, and serve as a hard-protecting shell to the development of the seed. Specifically, SiO_2_ is concentrated on the prickles and papillae cells (indicated by red arrows in Figure 15B–C) on the glume and lemma^8^ structures. Cells found in the leaf accumulate silica in their walls as well. Since leaves are remarkably involved in plant transpiration, the evaporation of water promotes the condensation of silica in these structures. Figure 15D–E illustrates the cross-section micrographs of a leaf, showing silica bodies in the leaf’s epidermis and tip, respectively (indicated by red arrow). The deposition of silica also extends to the root’s region, the first organ exposed to Si(OH)_4_ present in the soil. In Figure 15F–G, one can observe the presence of larger aggregates of silica along the cell walls of the root’s endodermis (indicated by red arrows).

**Figure 16:**
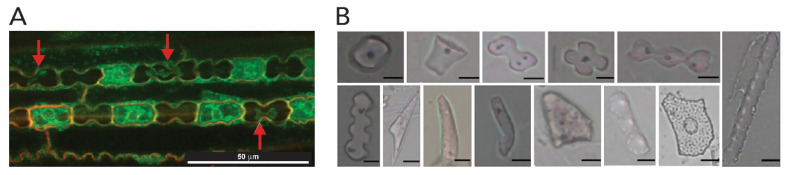
Silica bodies in plants. (**A**) Fluorescence microscopy of silica cells. Arrows indicates extra-membranous silica deposition. (**B**) Some examples of phytoliths from herbaceous plants. Scale bar = 10 mm. Image A is reproduced from [115] and image B is reproduced from [116], both with permission from John Wiley and Sons.

Once silicic species are taken up by the roots, they are transported to and deposited inside the cells, in the cell walls (as exemplified before) and in extracellular spaces of stems and leaves as hydrous amorphous silica particles, so-called phytoliths (Figure 16). Unlike for diatoms and sponges where silica deposition is much better understood, there have been contrasting hypotheses to explain silica deposition in plants, in particular to understand the formation of phytoliths [113]. One of the most accepted hypotheses is the passive silicification that relies on the silica deposition by organ transpiration, which implies that this is a spontaneous process resulting from auto-condensation of Si(OH)_4_ as water evaporates. Another hypothesis involves an enzymatic catalysis, or even a combination of both approaches [112].

In the passive silicification in plants, soluble silica in the soil is usually present as silicic acid Si(OH)_4_ and it is taken up by the roots and transported through the xylem (the tissue responsible for transporting water and dissolved compounds from roots to stems and leaves). Silicic acid species (e.g., Si(OH)_4_, (OH)_3_Si-O-Si-(OH)_3_, [SiO_n/2_(OH)_4-n_]_m_) are transported through the plant to a deposition site (the cell wall, the cell lumen and in intercellular and extracellular spaces) where the silica concentration is raised to the extent that it precipitates out to form a solid hydrated silica body (Figure 16A) [109,114]. The silica bodies, the phytoliths, exhibit interesting morphologies as illustrated in Figure 16B. Although amorphous silica has only short-range order, cellulose and other carbohydrates are ordered structures, and therefore impart a certain shape on the silica that is deposited [3].

One of the proposed roles of silica in plants is to crosslink their cell wall-forming polymers, and thereby increasing their compressive strength [117]. Silicification is preferentially chosen in certain species for improving mechanical properties of some tissues. For improving mechanical properties, these plants could technically ‘choose’ between inorganic SiO_2_ fortification, and organic lignin fortification. While silicification comes at lower metabolic cost than lignin formation, the water repellency of SiO_2_ cannot compare to that of lignin, and thus an optimal property compromise is generated by a composite of both [111]. Among other functions, it has been suggested that phytoliths serve a variety of purposes, including (i) to keep the leaf blades erect, thus allowing light to reach the lower leaves and hence increasing the extent of photosynthesis; (ii) to protect from UV radiation; (iii) to promote structural rigidity, i.e., preventing it from falling over and giving mechanical strength and rigidity to leaves; and (iv) to act as a defense against both vertebrate and invertebrate herbivores by increasing the abrasiveness of grass leaves [3,114,118,119,120]. Note that different phytolith types present in various plant species have been compiled in a recent review by Sharma and coworkers [121].

**Figure 17:**
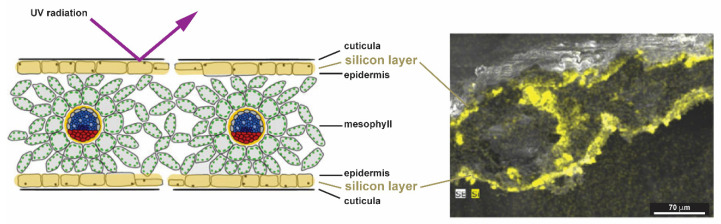
Scheme of a leaf cross section and distribution of silica (colored as yellow) in the leaf cross-section of a dried leaf of Phragmites australis (a type of common reed) revealed by SEM equipped with an element detector EDR. Reproduced from [119] with permission from Springer Nature.

Silicified structures are also largely found in grasses grown for their edible grains, such as cereals (e.g., rice, oat and wheat) [114,122]. Rice, for example, is the plant accumulating the highest known amounts of silicon. Silicon is most abundant as cuticle-silica layer in the inflorescence husk [12,123]. Cuticle-silica has important roles as a component for enhancing mechanical properties, stability, and as a physical barrier [124]. It was shown for seedlings of soybeans, wheat, and reed, that an amorphous silica double layer near the epidermis reduces stress induced by UV-radiation, acting like a glassy barrier and reducing the UV transmission (Figure 17) [119].

**Figure 18:**
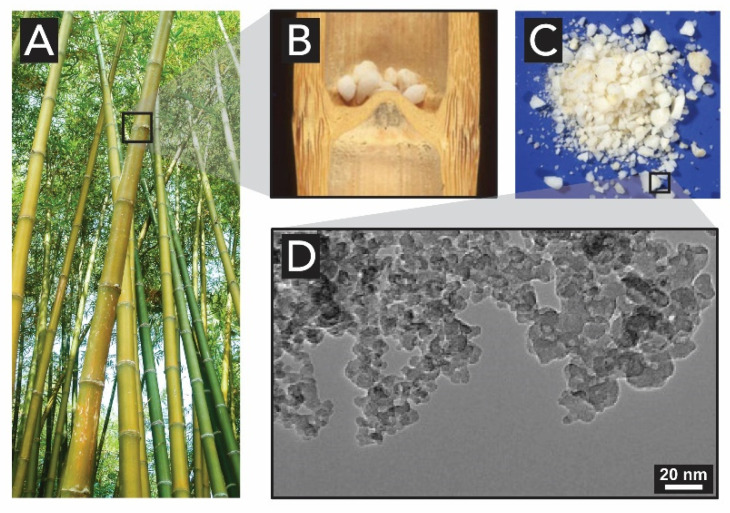
Bamboo plants and their silica content. (**A**) Bamboo tree forest. (**B**) Close look on tabashir granulae inside the pit cavity of old culms. (**C**) Image of tabashir particles collected from old culms. (**D**) TEM image of biogenic hydrated SiO_2_ particles. Images A–C are reproduced from [126] with permission from Springer Nature. Image D is adapted from [127] with permission from Elsevier.

The hardness and stiffness of bamboo (Figure 18A) is due to the presence of intracellular silica in the fiber structure. Some species of bamboo contain extracellular silica in the hollow stems as a gelatinous mass known as ‘tabashir’. It can be isolated from the plant tissues and appears to be the residue of the watery liquid also found in the hollow internodes of the plant. Once the bamboo ages and the gelatinous mass become solid, it deposits in the pit cavity as loose grains (Figure 18B). The reason for the origin of such enormous amounts of silica remains to date still unclear. Sanders et al. [125] were the first to study these structures in detail and showed that the tabashir particles (Figure 18C) were nearly spherical amorphous silica particles of ~100 Å, that give tabashir a milky appearance.

Silica, isolated from the plant tissues by ashing or by acid digestion, has often been identified as opal-type (i.e., amorphous SiO_2_) by optical and electron microscopy, and X-ray diffraction techniques. Whether in plants or animals, at the microscopic level, biogenic silica is always considered to be amorphous, showing no order beyond 10 Å, or approximately three Si-O-Si units. Nevertheless, Sterling [128] identified the first examples of crystalline silica—together with amorphous silica - in strawberry leaves and tabashir. In tabashir, the crystalline parts of SiO_2_ could be ascribed to low tridymite and α-cristobalite through X-ray diffraction analysis, and to quartz in strawberry leaves. Reasons for the appearance of crystalline forms of SiO_2_ in these plants are yet unclear and, to the best of our knowledge, further observations have not been reported.

**Figure 19:**
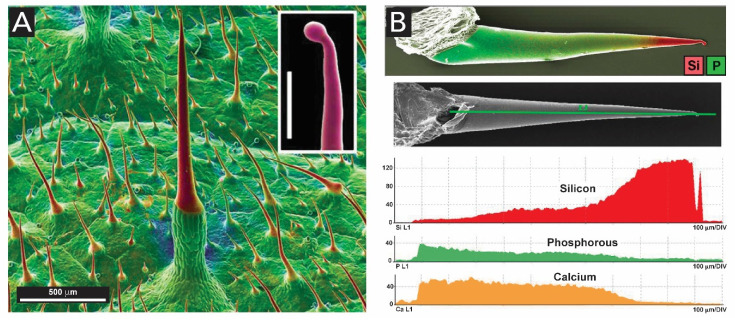
Morphology and composition of stinging hairs of *Urticaceae*. (**A**) Cryo-SEM micrographs of typical stinging hairs on leaves of *Urtica dioica*. Insert: silicified bulbous tip of stinging hair (red: high silicon content; green: organic compounds only; scale bars: 50 μm). (**B**) Element-mapping images. Line scans display concentration profiles from tip to base (green scale bar: 300 μm). Adapted from [130] with permission from John Wiley and Sons.

Silica bodies can also be present for defense, making some plants distasteful to herbivores or give their tissues a prickly texture. Typical examples are cacti that contain barbs, and rose stems with sharp hooks. The hairs that are responsible for the stinging caused by stinging nettles, the so-called trichomes, are made of fine hollow needles of silica [129]. Studies on the mineralization within *Urticaceae*, a herbaceous plant which has jagged leaves covered with stinging hairs (Figure 19A) show that SiO_2_ is dominant in all stinging hair tips (Figure 19B) over any other parts of the structure [130]. These hairs are sharp enough to penetrate skin and inject a mixture of inflammatory substances, including, e.g., histamine, acetylcholine and formic acid [131].

**Figure 20:**
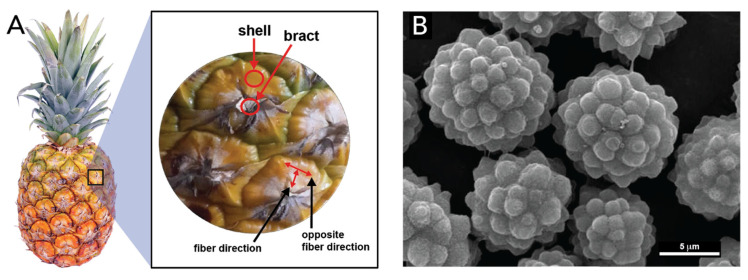
Pineapple parts and their silica microparticles. (**A**) Pineapple peel detail. (**B**) SEM image of isolated SiO_2_ microparticles found in the bracts of the pineapple peel exocarp (shell) after extraction. Adapted from [132] under a Creative Commons Attribution 4.0 International License (CC BY 4.0).

Very recently, silica structures were identified in pineapples bracts and shells (Figure 20) [132]. Their shells feature rosette-like microparticles (Figure 20B) that were identified as biogenic silica, responsible for giving support and mechanical resistance to the shell structure, among supposedly other, yet unexplored, functions.

### 2.2. Aluminum Oxide and Germanium Oxide

One might expect that, during the development of a plant, elements other than silicon and oxygen will be incorporated into the structures of phytoliths structures. Indeed, so far 24 different elements were detected in plant phytoliths [133].

As for silicon, aluminum can also be associated with phytoliths of plants. However, the accumulation of aluminum and silicon appear to be mutually exclusive. High silicon accumulators (e.g., rice) take up little aluminum, while aluminum accumulator plants take up little silicon [134]. Only a very small number of Al-accumulators is known to date. A small number of woody species (e.g., conifers) accumulate aluminum in the form of aluminosilicates in their phytolith structures [135]. Although aluminum is an abundant element, it is not essential for biological systems and their processes. The abundance of aluminum (~8% in the crust compared to ~27% for Si) and the inexistence of a biological system associated with it are intriguing. One possible explanation is that most of Al released by the weathering of aluminosilicates present in the soil is actually taken up geologically, to form phyllosilicates. The higher the Si concentration in soil, the more Al-phyllosilicates form over aluminum oxides. Furthermore, the complexation of Al^3+^ by organic matter prevents aluminum oxide precipitation [9,136].

Another M_x_O_y_ that one might expect to find in organisms is germanium oxide (germania) due to its chemical similarity with silica (yet at low abundance in the crust: 0.00014%). Trace quantities of Ge can be found within the silica structures made by diatoms or sponges; however, GeO_2_ cannot be found as part of any living structure. In fact, elevated concentrations of germanic acid have been observed to poison diatoms and sponges, and to produce distortions in the silica structures formed by these organisms [137].

### 2.3. Iron Oxides

Iron oxides are found in organisms to accumulate iron for future metabolic needs, serving for strengthening and hardening of tissues, intracellular iron storage, detoxification, and sensing of magnetic fields [33]. Examples of biogenic iron oxides are found in bacteria, algae, bees, mollusks, fish, pigeons and mammals [138].

**Figure 21:**
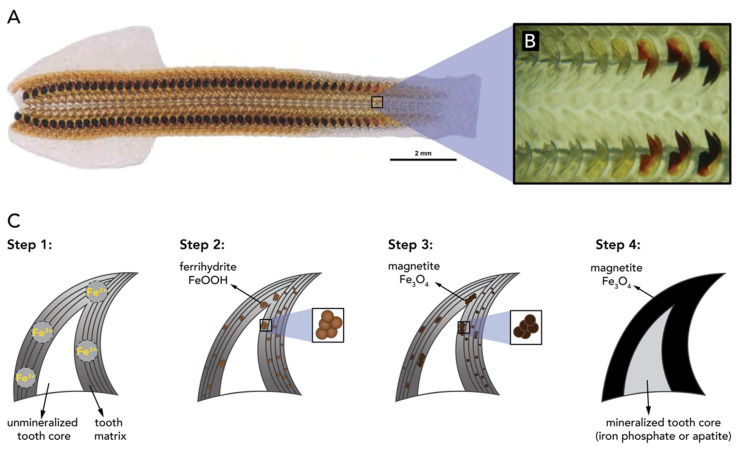
Chiton teeth and their formation. (**A**) Light micrograph of the radula of the chiton *Acanthopleura gaimardi*. (**B**) Detail of progressive stages of radular tooth development. (**C**) Scheme of the hypothesized mechanism of iron mineral formation (ferrihydrite and magnetite) in chiton teeth. Step 1: Organic tooth matrix around the unmineralized tooth core, which adsorbed Fe^3+^; Step 2: Fe^3+^ is first transformed into ferrihydrite; Step 3: conversion of ferrihydrite into magnetite; Step 4: mineralization of magnetite shell proceeds and the tooth core is also mineralized to phosphates. Image A reproduced from [142] by Brooker and Shaw, 2012, reproduced under the terms of the Creative Commons Attribution 3.0 License. Image B is reproduced from [141] with permission from Springer Nature.

Ferrihydrite (5Fe_2_O_3_·9H_2_O), lepidocrocite (γ-FeOOH), goethite (α-FeOOH) and ferric oxide (Fe_2_O_3_·nH_2_O) have been found in animal species. The major iron oxide present in living nature is however magnetite (Fe_3_O_4_), which is ferrimagnetic and has a hardness of 6.5 on the Mohs scale [33]. The first discovery of magnetic Fe_3_O_4_ in biological systems was in the teeth (denticles) of chiton mollusks, a species of marine mollusks from the kingdom *animalia* and phylum *mollusca* (Figure 21A,B) [68]. The high iron concentration in chiton denticles allows them to abrade particles of algae from the substrate they live on, which they ingest [139].

It was shown that the mineralization processes of the teeth in *Cryptochiton stelleri*, a specimen of chiton, features the precipitation of iron minerals inside an organic matrix (Figure 21C). As the teeth mature, the ferric mineral ferrihydrite is transformed to magnetite (Fe_3_O_4_) [140]. Recently, it was hypothesized that the synthesis of magnetite in chiton teeth occurs through the following procedure (Figure 21C): Initially, soluble ferric ions are transported into the tooth matrix, made of α-chitin and proteins (e.g., ferritin), through the tooth surface (step 1). The adsorbed hydrated iron species precipitate as ferrihydrite with the aid of ferritin proteins (step 2). Then, ferrihydrite is converted to magnetite via a solid-state transformation and magnetite crystals grow along the chitin fiber until the space between particles and chitin fibers is completely filled (step 3). In the end, the tooth core region of apatite (Ca_5_(PO_4_)_3_(OH,F,Cl)) or iron phosphate mineralizes to complete the tooth formation (step 4). This mechanistic hypothesis was based on a combination of structural analysis and proteomics [141].

**Figure 22:**
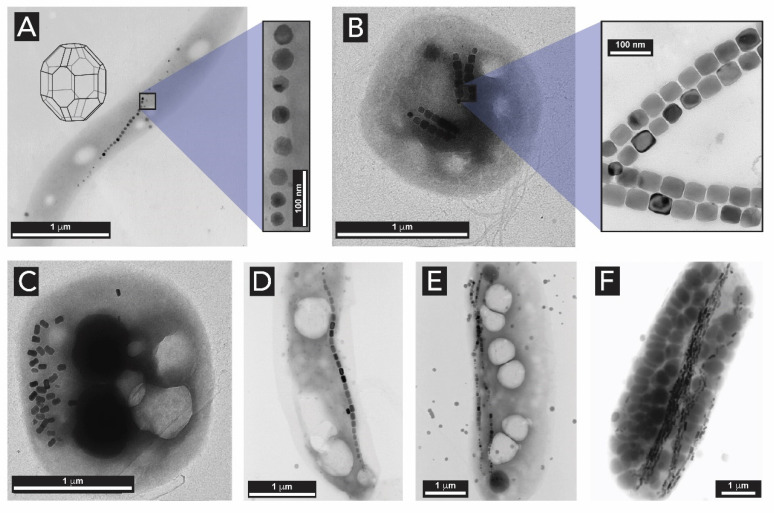
TEM images of different magnetotactic bacteria (Gram-negative type) and magnetosome chains (black dots). (**A**) A spirillum with a single chain of polyhedral magnetosomes. Inset illustrates a model of the particles. (**B**) A coccus with two double chains of slightly elongated prismatic magnetosomes. (**C**) A coccus with clustered, elongated magnetosomes. (**D**) A vibrio with elongated prismatic and cubo octahedral magnetosomes arranged into a single chain. (**E**) A vibrio with two chains, and (**F**) a rod-shaped bacterium with bullet-shaped magnetosomes arranged into several parallel chains. Adapted from [1] with permission from the American Chemical Society.

After the initial discovery of biogenic magnetic iron oxide in the teeth of chitons by Lowenstam in the 1970s [140], certain bacteria were discovered to contain chains of single crystalline magnetite particles that act as small magnets and allow for these bacteria to align along the magnetic field of the Earth [45]. The iron oxide bearing bacteria are named ‘magnetotactic bacteria’. In these, uniformly sized particles of magnetite are often arranged in different chains and their morphologies include rods, vibrios, spirilla, cocci, and ovoid bacteria (Figure 22). Nothe the following definition: Vibrio: a genus of Gram-negative bacteria, possessing a curved-rod (comma) shape. Spirilla: Gram-negative bacteria with a helical or spiral shape. Cocci: any bacterium that has a spherical shape. Magnetotactic bacteria are the most intensely studied types of biogenic iron oxides. A list with all the isolated and characterized species of magnetotactic bacteria is reported in [143]. Blakemore was the first to describe the presence of iron-rich particles within membrane vesicles [45]. In these systems, the particles are enclosed in membrane vesicles, forming structures known as a magnetosomes and enabling the organism with a behavior known as magnetotaxis (the property of movement along magnetic fields) [144].

The mineralization of magnetic particles in magnetotactic bacteria is still under investigation. Based on Mössbauer spectroscopy and biochemical analyses, it was suggested that for bacterial magnetite formation, Fe^3+^ ions are taken up from the environment and reduced intracellularly. Then, mineral precipitation occurs within the magnetosome vesicles, possibly by a partial reduction in hydrated ferric oxide [145,146,147].

**Figure 23:**
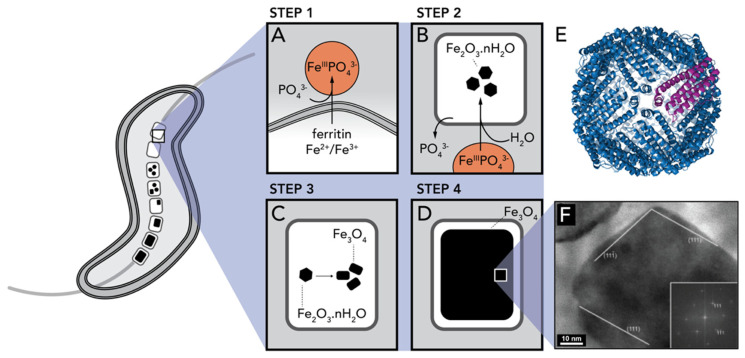
Model of iron uptake and magnetite formation mechanism in magnetotactic bacteria. (**A**) Step 1: Ferritin and iron ions are transported through the cytoplasmatic membrane of the bacteria and a phosphate-rich Fe^III^ hydroxide phase is formed. (**B**) Step 2: Magnetite biomineralization starts by transport of iron ions and ferritin into the magnetosome vesicles where Fe^2+^ and Fe^3+^ ions coprecipitate to a ferrihydrite-like intermediate. (**C**) Step 3: Ferrihydrite is converted to magnetite, and (**D**) Step 4: it grows fully formed magnetosomes. (**E**) Ferritin protein complex. (**F**) HR-TEM image of the magnetosome from magnetotactic *Gammaproteobacteria strain SS-5*. Mechanism adapted from [151] with permission from John Wiley and Sons. Image (**E**) authored by Vossmann, and reproduced under the terms of the GNU General Public License. Image (**F**) by Pósfai, Lefèvre, Trubitsyn, Bazylinski and Frankel, 2013, reproduced under the terms of the Creative Commons Attribution License (CC BY).

Using a combination of Mössbauer spectroscopy and electron microscopy, Schuler et al. examined the cellular iron uptake in magnetotactic bacteria in detail, and hypothesized the following mechanism of formation of biogenic Fe_3_O_4_ (Figure 23): First, the bacteria take up either ferric iron (Fe^3+^) or ferrous iron (Fe^2+^) (Step 1) and convert it intracellularly to a ferrous high-spin species (Step 2). These iron species remain associated with the cytoplasmic membrane until mineralization (Step 3), when the magnetite crystals (Figure 23F) grow to entirely fill the magnetosome compartment (Step 4) [148]. Their analysis indicated that ferritin might play an important role in the coprecipitation of ferric and ferrous iron ions at the magnetosome membrane, although its exact role has not been fully elucidated. Ferritin is a hollow spherical protein bearing iron ions in its cavity (Figure 23E). The iron core of the protein is described as a mineral of the ferrihydrite type, and it is considered essential for the iron storage of various organisms, including humans [149]. Ferritin is commonly considered a crucial precursor in the synthesis of magnetite in diverse biological systems [150].

The discovery of the existence of inorganic magnetic materials in bacteria prompted investigations of their existence and role in the orientation of higher animals. It was observed that some migratory animals appear to be sensitive to magnetic fields. With further studies, the occurrence of magnetic particles was reported in different tissues of several animals, e.g., rodents [152], bats [153], honeybees [2], pigeons [154,155], fishes [156], and marine mammals [157]. Even in humans, magnetic iron oxide nanoparticles have been identified in different tissues from the brain, heart, liver, and spleen, and it is deemed possible that they are of biogenic origin [158,159,160,161].

**Figure 24:**
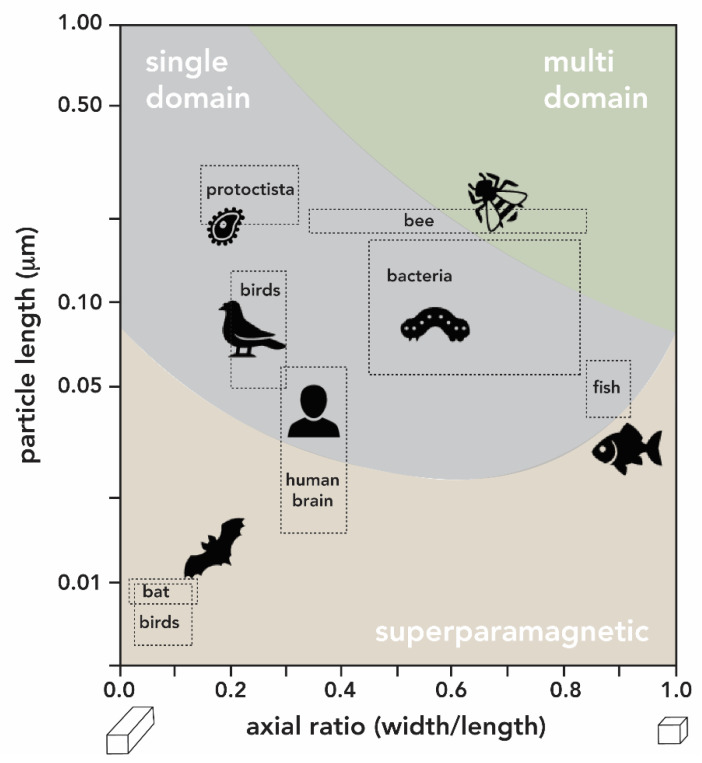
Magnetite in living systems subdivided as a function of particle size and magnetic behavior (single domain, multi domain or superparamagnetic). Adapted from [163] with permission from Springer Nature. Information on additional species was compiled from [164].

The size and morphology of the magnetite particles are species-specific (Figure 24). Biogenic magnetite particles can be classified according to their size and magnetic properties into: (*i*) large particles (>1.0 μm) with their magnetic moments largely canceling each other out (known as multi domains); (*ii*) particles in the range between about 0.05-1.0 μm featuring a stable magnetic moment, and acting as tiny permanent magnets (known as single domain); and (*iii*) superparamagnetic particles (3–5 nm), with magnetic moments that can be relatively easily aligned by an external magnetic field [162,163]. It is assumed that the magnetic crystal sizes are determined by spatial constraints exerted by the local environment (such as the magnetosome vesicle size). Furthermore, the biomineralization of the ferrihydrite core of ferritin is considered a possible pathway for the formation of larger magnetite particles [150]. Thus, growth of Fe_3_O_4_ particles in living systems might be regulated to generate the species-specific sizes that are effective for the distinct purpose of the species. One of the limitations in these studies relies on the magnetite nanoparticles’ sizes: given that iron is common in several physiological processes, is extremely difficult (with the current analytical tools) to differentiate between magnetic particles and the iron compounds used in other metabolic activities [164].

### 2.4. Manganese Oxides

Manganese oxides (including oxides, hydroxides, and oxyhydroxides) are important minerals in biogeochemical cycles. The oxidation of soluble Mn^2+^ species to insoluble Mn^3+/4+^ oxides is an environmentally important process since MnO_2_ can oxidize a variety of organic and inorganic compounds, scavenge many metals from the environment (e.g., Cu, Co, Cd, Zn, Ni, and Pb), and serve as electron acceptor for anaerobic (Anaerobic respiration is the respiration using electron acceptors other than O_2_)respiration of certain living organisms [165].

In the environment, the catalytic role is filled by a class of microorganisms referred to as ‘manganese oxidizers‘. To date, the only species bearing biogenic Mn oxides are Mn-oxidizing/reducing bacteria and fungi [166], and the involved Mn oxide formation process is largely unresolved. Through laboratory culture experiments, it has been proposed that Mn oxidation and reduction is mainly mediated by bacteria. Over the years, Mn-oxidizing bacteria have been isolated and identified from a wide variety of environments, including both the oceans and freshwaters, sediments, and hydrothermal vents [21,166,167,168,169]. The mechanism of Mn^2+^ oxidation and oxide formation by microorganisms is not well established, however, several studies evidence that certain enzymes (multicopper oxidases), appear to be involved in Mn oxidation in all of these organisms [170,171,172,173].

Manganese oxidizing bacteria species are reported to produce mixed-valence Mn oxides with structures corresponding to the minerals todorokite or birnessite [174,175,176,177]. The todorokite-type tunnel and birnessite-type layered structures found in biogenic manganese oxides are structurally closely related to each other. Synthetically speaking, birnessite can be transformed easily into todorokite via hydrothermal treatment and cation exchange [178,179].

**Figure 25:**
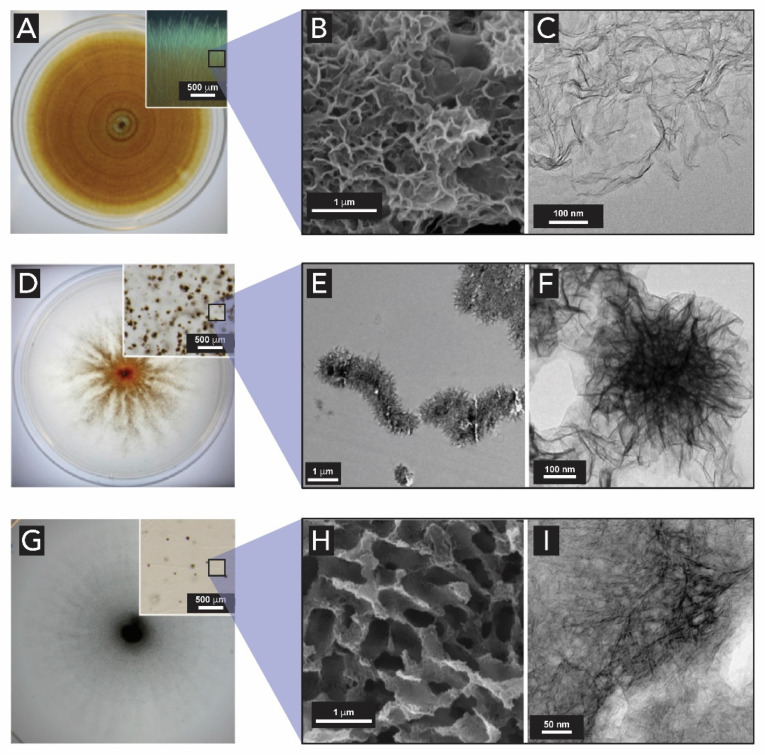
Optical microscopy, SEM, and HR-TEM images of Mn oxides produced by *Plectosphaerella cucumerina* DS2psM2a2 (**A**–**C**), *Stagonospora sp.* SRC1lsM3a (**D**–**F**) and *Acremonium strictum* DS1bioAY4a (**G**–**I**) growing radially outward from the inoculation point in the center of the petri dishes. (**A**,**D**,**G**) Optical micrographs of thin sheets of Mn oxides on their surface. (**B**,**E**,**H**) SEM images of the Mn oxides particles produced by the fungi. (**C**,**F**,**I**) HR-TEM images showing the morphology of Mn oxides. Adapted from [185] with permission from Elsevier.

Despite bacteria being the dominant Mn-oxidizing organisms, Mn-oxidizing fungi have also been observed in extreme environments such as deep-sea ferro-manganese crusts [180], and in terrestrial environments [181,182]. In some surface and terrestrial environments, fungal Mn oxidation is more important than bacterial oxidation [183]. Despite the lack of a universally accepted model, some experimental work tries to expand the knowledge on biogenic Mn_x_O_y_ in diverse species of Mn-oxidizing fungi by characterizing them through microscopic and spectroscopic techniques [182,184,185]. The studies reveal that, depending on the species of Mn-oxidizing fungi, different Mn_x_O_y_ sizes and morphologies, and chemical structures are found (Figure 25). For example, the Mn oxides produced by *Plectosphaerella cucumerina* type (Figure 25A–C) have a typical nanoparticulate, plate-like morphology, as the ones observed for Mn(II)-oxidizing bacteria. Mn oxides observed in *Stagonospora sp.* type (Figure 25D–F) form densely packed filaments of Mn oxides. The third example, the *Acremonium strictum* (Figure 25G–I) exhibits rather poorly defined morphologies and the Mn oxide conglomerate presents a honeycomb-like appearance [185].

Petkvok et al. showed that Mn-oxidizing fungi prefer a less moist environment, oxidize Mn ions relatively slowly, and are prone to produce todorokite particles of higher crystallinity (almost defect-free), compared to Mn-oxidizing bacteria [184]. Santelli et al. have shown that the fungal organic matrices play an important role in organizing Mn_x_O_y_ structures in order to decrease thermodynamic barriers for precipitation of more crystalline phases like todorokite [185].

Mn oxides precipitated by the Mn-oxidizing organisms accumulate on the extracellular matrix of the organisms for both bacteria and fungi. However, to date, the physiological reasons behind the Mn oxidation is yet unclear. Some authors suggest that one of the reasons might be to harvest energy for their growth [13,166]. Further hypotheses are that the resulting Mn_x_O_y_ can increase the accessibility of organic nutrients and protect the microorganism from potentially toxic compounds, reactive oxygen species and UV radiation stress [186,187]. Zerfaß et al. have recently shown that the Mn oxides in *Roseobacter sp. strain AzwK-3b*, are involved in the catalysis of nitrites (known as bacteria inhibitors) into nontoxic nitrate compounds, which thus protects bacteria against nitrite [188].

Although biogenic Mn_x_O_y_ are only produced by simple organisms such as bacteria and fungi, it has been found that they play a role in the symbiosis with more complex living systems. For example, certain plants bear Mn-oxidizing bacteria or fungi within their tissues. Here, the microorganisms support the plant growth and development. Recent studies on wetland plant species (e.g., *Egeria densa* or *Suaeda salsa pall*) have revealed that their associated bacteria have Mn-oxidizing activities, enabling the synthesis of Mn_x_O_y_ biofilms on the plant roots’ surfaces [189,190,191]. The appearance of Mn oxides as plaques on the surface and in the pit (a tissue in the stems of vascular plants, which store and transport nutrients throughout the plant) of these wetland plants enhance further precipitation of Mn oxides on the plant roots, possibly serving as an oxidant of heavy metals and pollutants and as a scavenger of trace nutrients to sustain the growth of these plants in saline and heavy metal-contaminated wetlands.

## 3. Biomimetics of Biogenic Metal Oxide Structures and Further Inspirations and Perspectives for Materials Synthesis

Hierarchical and functional inorganic M_x_O_y_ structures are found in living organisms of all the kingdoms, from simple bacteria or fungi to more complex plants and animals. Clearly, these structures present an intriguing source of inspiration for novel synthetic pathways for the production of materials, but also for novel synthetic materials.

One approach in the biomimicry of biogenic M_x_O_y_ is to investigate the biomolecules that organisms use for controlling M_x_O_y_ precipitation for synthetic M_x_O_y_ generation, as well as to derive synthetic variants of these biomolecules. This includes to extract, isolate, characterize, and understand the role of biomolecules involved in the mineralization. To date, several examples of successful in vitro mineralization employing proteins and peptides extracted from organisms that biosynthesize M_x_O_y_ have been reported. As several reviews have summarized both the biomimetics of natural M_x_O_y_ assemblies [14,15,192,193,194], and biomimetics approaches to magnetic materials [195], we will not go into further detail here.

**Figure 26:**
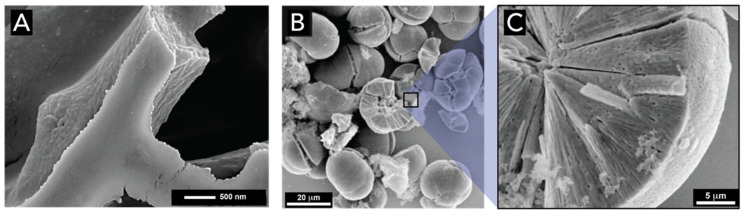
Biomimetic synthesis of titanium dioxide. SEM micrographs of (**A**) TiO_2_ product formed by catalysis with purified sponge silicatein filaments and (**B**) titania particles made by diatom silaffins and zoom in (**C**). Scale bars: (A) 500 nm, (B) 20 μm, (C) 5 μm. Image A reproduced from [197] with permission from American Chemical Society. Images in B are adapted from [196] with permission from John Wiley and Sons.

Biomolecules mediating biogenic M_x_O_y_ formation have also been utilized to synthesize materials beyond those produced by the original living organism. For instance, current approaches include the in vitro use of proteins from diatoms (so-called silaffins) [196], or silicatein filaments from sponges [197], to obtain TiO_2_ nanostructures (Figure 26). It is shown that both types of proteins, although from different species, are capable of catalyzing and templating the hydrolysis and condensation of titanium alkoxides to form TiO_2_. Most interestingly, TiO_2_ obtained from silaffin-mediated synthesis generates spherical, diatom-esque morphologies. Furthermore, proteins and peptides extracted from diatoms were used in the in vitro mineralization of new amorphous silica structures [198], and gallium oxide [199]. In addition, other proteins have been used to generate M_x_O_y_ that are not found in living organisms, such as germanium oxide [200], cobalt oxide [201], zinc oxide [202], multi component oxides [203], and perovskite-type oxides [204].

The use of compounds of biological origin extracted from organisms for materials synthesis has, however, some drawbacks. The species may be difficult to obtain, many require specialized facilities for their growth, and they yield limited quantities of the desired biomolecules. Furthermore, the number of biomineralization proteins that have been identified (and sequenced) to date is still quite limited.

Aside biomimicry of biological M_x_O_y_ synthesis, biogenic M_x_O_y_ can also serve as inspiration for inorganic-organic composites and hybrids. Note that the term ‘composite’ was historically used for larger structures not featuring strong bonding between matrix and dispersed phase, while ‘hybrid’ was reserved for strongly bonded inorganic-organic materials with phase domains in colloidal size range (1-1000 nm). Nowadays, both terms are often used interchangeably. A first learning that one can draw from biogenic M_x_O_y_—which, with the exception of Fe oxides, are composites of Fe_x_O_y_ intricately connected to biomolecules—is which types of organic compounds M_x_O_y_ are likely (as observed in nature) to be compatible with. The interfaces between inorganic and organic phases in composites/hybrids are both the loci that have the strongest impact on synergistic properties and also the loci of materials failure [205]. From the distinct combinations of M_x_O_y_ with, e.g., certain polysaccharides or peptides and proteins in biogenic M_x_O_y_ materials, one can infer with high certainty that a combination of closely related materials will also exhibit good compatibility. Indeed, the recent developments in the field of cross-linked metaloxides particles uses polysaccharides cross-linkers such as alginate [206]. Several examples of so-called ‘extreme biomimetic’ synthesis have exploited the generation of M_x_O_y_-polysaccharide composites by extremophile organisms [6], to generate, e.g., chitin/SiO_2_ [207], chitin/ZnO [208], and chitin/ZrO_2_ [209]. Furthermore, biogenic M_x_O_y_ in living organisms are never (as from all reported examples to date) biosynthesized concomitantly with the biomolecules one finds in the final M_x_O_y_ composites. This allows for the conclusion that the truly simultaneous synthesis of organic and inorganic components is likely to yield radically different synthetic hybrid materials than what is found in nature. Indeed, rare examples of truly concomitant synthesis yield fascinating materials structures and properties, e.g., with respect to crystallinity [210]. Alternatively, one might also conclude that the intricate M_x_O_y_ composites found in nature cannot be synthetically generated through simultaneous synthesis, but actually require the presence of biomolecules (with, e.g., the templating and structure-directing effects), before M_x_O_y_ is precipitated.

Instead of gathering inspiration from the actual materials found in biominerals, one might also look at the types of reactions that lead to them in living organisms, and apply them to chemically quite different compounds that, however, form by similar mechanisms. We have recently explored such strategies that are however inspired by materials formation in non-living natural systems, i.e., so-called ‘geomimetics’ [6]. Prompted through the fact that metal oxides, such as the here intensively discussed SiO_2_, but also various silicates and all natural zeolites, are in the Earth’s crust forming through polycondensation (i.e., the addition of small molecules or ions to one another accompanied by the release of one or several H_2_O molecules at each addition step) under hydrothermal conditions (i.e., in liquid H_2_O at *T* > 100 °C and *p* > 1 bar), we have explored the formation of organic molecules under hydrothermal conditions [211]. Through geomimetic hydrothermal syntheses, several types of compounds (even highly aromatic) could to date be generated, including polyimides [212,213,214,215,216], low-molecular weight imide and imidazole dyes [217,218], polybenzimidazoles and pyrrone polymers [219], and even simultaneously synthesized polyimide-SiO_2_ hybrid materials [210]. In analogy, it is conceivable that the fact that M_x_O_y_ are formed in living organisms under a given species-specific set of formation conditions (aqueous environment, moderate temperatures, a certain pH range, the presence of certain additives), allows for synthesizing organic materials that mechanistically form by closely related reactions (again, e.g., condensations) under similar conditions.

Finally, especially in times of increasing awareness of the scarceness of natural resources and several other aspects of sustainability, biogenic M_x_O_y_ can actually be used as resources. For instance, there are several reports on the use of rice rusk (which is with up to 20% hydrated SiO_2_ in dry weight highly silicified) as resources for the generation of silsesquioxane building blocks [220], and SiO_2_ nanoparticles [221,222]. Along these lines, it is also conceivable to generate advanced materials through the pyrolysis of biogenic M_x_O_y_. Similar approaches have been reported for heavy metal nanoparticles supported on carbon materials through the pyrolysis of thale cress plants grown on Pd-enriched soils. Clark and coworkers could generate carbon-supported Pd^0^-nanoparticles and show the effectiveness of these materials as catalysts [223].

## 4. Conclusions

With this review, we have provided an overview of the metal oxides (SiO_2_, iron and manganese oxides) found in the five kingdoms of living nature. It has become clear that there are several functional biogenic M_x_O_y_ structures, spanning, e.g., from amorphous to crystalline, presenting magnetic and/or oxidative properties, that can be generated biologically. To date, for all known biogenic M_x_O_y_, we have summarized the current understanding of (*i*) where their precursors come from, (*ii*) how the precursors are taken up by the organisms, (*iii*) how the resulting M_x_O_y_ are precipitated, and (*iv*) which structures for which purposes result. Examples of biomimetic approaches for M_x_O_y_ were shown to demonstrate the ongoing efforts in biomineralization research. Metal oxides in living nature are incredibly complex and understanding their architectural design principles can still provide a large body of inspiration for biologists, chemists, and material scientists alike to create a plethora of novel materials.
